# Draft Genome Sequence of *Brevibacillus choshinensis* HPD52^T^ (DSM 8552), a Bacterial Host for Efficient Expression of Heterologous Proteins

**DOI:** 10.1128/genomeA.01688-15

**Published:** 2016-02-04

**Authors:** Jie-ping Wang, Bo Liu, Guo-hong Liu, Jian-mei Che, Zheng Chen, Meichun Chen, Huai Shi

**Affiliations:** Agricultural Bio-Resources Research Institute, Fujian Academy of Agricultural Sciences, Fuzhou, Fujian, China

## Abstract

*Brevibacillus choshinensis* HPD52^T^ (DSM 8552) is a Gram-positive, spore-forming, and protein-producing bacterium. Here, we report the 6.28-Mb draft genome sequence of *B. choshinensis* HPD52^T^, which will promote its application and provide useful information for genomic taxonomy and phylogenomics of *Bacillus*-like bacteria.

## GENOME ANNOUNCEMENT

The protein-producing strain HPD52^T^ (DSM 8552) was initially assigned as a member of *Bacillus brevis* (now *Brevibacillus brevis*); however, it was identified as a unique species and named *Bacillus choshinensis* sp. nov. in 1993 (1). Subsequently, *B. choshinensis* was reclassified as *Brevibacillus choshinensis* comb. nov. in 1996 (2). It is of great significance that *B. choshinensis* is an effective Gram-positive bacterial expression system for secretory proteins, due to its strong capacity to secrete a large amount of proteins (approximately 30 g/liter) (3–5). Because of the application prospects and lack of available genomic information of *B. choshinensis*, strain HPD52^T^ was selected as one of the research objects in our “Genome sequencing project for genomic taxonomy and phylogenomics of *Bacillus*-like bacteria” (J.-P. Wang, Bo Liu, G.-H., Liu, C.-B. Ge, Q.-Q. Chen, J.-M. Che, and D.-J. Chen, unpublished data). Here, we present the first draft genome sequence of *B. choshinensis*.

The genome sequencing of *B. choshinensis* HPD52^T^ (DSM 8552) was performed via the Illumina HiSeq 2500 system. Two DNA libraries with insert sizes of 262 and 4,920 bp were constructed and sequenced. After filtering the 1.22 Gb of raw data, 1.15 Gb of clean data were obtained, providing approximately 184-fold coverage. The reads were assembled via the SOAP*denovo* software version 1.05 (6), using a key parameter K setting of 68. Through the data assembly, 16 scaffolds with a total length of 6,279,095 bp were obtained, and the scaffold *N*_50_ was 2,087,727 bp. The average length of the scaffolds was 392,443 bp, and the longest and shortest scaffolds were 2,198,769 bp and 687 bp, respectively. A total of 94.73% clean reads were aligned back to the genome, which covered 99.68% of the sequence.

Annotation of the genome was performed using the NCBI Prokaryotic Genome Annotation Pipeline (PGAP) (http://www.ncbi.nlm.nih.gov/genomes/static/Pipeline.html) utilizing the GeneMark, Glimmer, and tRNAscan-SE tools (7). A total of 6,371 genes were predicted, including 6,222 coding sequences (CDSs), 125 tRNAs, and 24 rRNA genes. There were 4,544 and 2,553 genes assigned to the COG and KEGG databases, respectively. The average DNA G+C content was 48.37%, agreeing with the value 48.2 mol% (1).

### Nucleotide sequence accession numbers.

This whole-genome shotgun project has been deposited at DDBJ/EMBL/GenBank under the accession number LJJB00000000. The version described in this paper is version LJJB01000000.
